# The Impact of International Research Collaborations on the Citation Metrics and the Scientific Potential of South American Palliative Care Research: Bibliometric Analysis

**DOI:** 10.5334/aogh.3158

**Published:** 2021-03-31

**Authors:** Crislaine de Lima, Bianca Sakamoto Ribeiro Paiva, Martins Fideles dos Santos Neto, David Hui, Pedro Emilio Perez-Cruz, Camilla Zimmermann, Eduardo Bruera, Carlos Eduardo Paiva

**Affiliations:** 1 Research Group on Palliative Care and Health-Related Quality of Life (GPQual), Barretos Cancer Hospital, Barretos, São Paulo, Brazil; 2 Oncology Graduate Program, Barretos Cancer Hospital, Barretos, São Paulo, Brazil; 3 Department of Palliative Care, Rehabilitation and Integrative Medicine, University of Texas, MD Anderson Cancer Center, Houston, Texas, USA; 4 Departamento Medicina Interna, Facultad de Medicina, Pontificia Universidad Católica de Chile, Santiago, Chile; 5 Department of Supportive Care, Princess Margaret Cancer Centre, University Health Network, Toronto, Ontario, Canada; 6 Department of Clinical Oncology, Breast and Gynecology Division, Barretos Cancer Hospital, Barretos, São Paulo, Brazil

## Abstract

**Background:**

Progress in palliative care (PC) requires scientific advances which could potentially be catalyzed by international research collaboration (IRC). It is currently not known how often IRC occurs with PC investigators in South America.

**Objectives:**

To evaluate the percentage of South America journal articles on PC involving IRCs and the impact of these collaborations on the scientific potential the studies and on their citations.

**Methods:**

This was a bibliometric analysis of studies published between January 1, 1998, and December 31, 2017. A search of Pubmed, Embase, Lilacs, and Web of Science (WOS) was performed using the terms “palliative care,” “hospice care,” “hospices” and “terminal care,” combined with the name of South America countries. The scientific potential was assessed by analyzing study design, characteristics of the journal and funding. IRCs were further subdivided in internal (within South America countries) and external (with countries outside South America).

**Findings:**

Of the 641 articles, 117 (18.2%) involved IRCs (internal: 18, 2.8%; external: 110, 17.2%). Articles with IRCs had higher median two-year citations in WOS (2 vs. 1, p < 0.001), Scopus (3 vs. 1, p < 0.001) and Google Scholar (4.5 vs. 2, p < 0.001) compared to articles without IRC. Moreover, they were more often funded (40.7% vs. 9.7%, p < 0.001), published in Pubmed-indexed (76.1% vs. 41.6%; p < 0.001) and in WOS-indexed (70.1% vs. 29.6%; p < 0.001) journals, and with study designs most often classified as clinical trial (5.1% vs. 1.0%; p = 0.002) and cohort (10.3% vs. 2.9%; p < 0.001) compared to articles without IRC.

**Conclusions:**

Studies with international research collaborations, both internal and external to South America, are more frequently cited and have characteristics with greater scientific potential than do studies without international collaborations.

## Background

The modern hospice movement, the current origin of palliative care (PC), originated in London (UK) in the 1960s, when Cicely Saunders, along with others, founded St. Christopher’s Hospice [1]. Subsequently, other hospitals emerged with the same goal, both in England and around the world [2]. In the United Kingdom, PC expansion reached a peak in the 1980s, but in the rest of Europe, the peak occurred a decade later. In the USA (United States of America), PC was included in the Medicare health program in 1982. This inclusion led to a considerable advance in the US PC network, which eventually increased from just over 1,500 services in 1985 to more than 4,000 in 2005 [2,3]. The start of PC in South America occurred only after 1980, and there has been an escalation in the number of specialized services and of health providers in the last three decades [4]; the growth rate was probably highest after the beginning of the current century. According with the “Palliative Care Atlas in Latin America” there were only 1.63 PC services per million people and half of them were located in Chile and Argentina [5].

The International Observatory on End-of-Life Care has analyzed in detail the presence and complexity of PC services around the world. Countries are classified into four groups: Group 1 (no PC activity); Group 2 (capacity building activity); Group 3 (isolated PC provision [3a] or generalized PC provision [3b]); and Group 4 (PC services in preliminary [4a] or advanced [4b] stage of integration). Among South America countries, Uruguay and Chile were ranked the highest (level 4a), followed by Argentina (3b) [6]. Additionally, in 2015, the Economist Intelligence Unit published, for the second time, a ranking of countries regarding quality of death. Among South America countries, the top ranked in 2015 were Chile (27th of 80) and Argentina (32nd of 80); Brazil, which was ranked 38th in 2011 (out of 40 countries evaluated), was ranked in 42nd place among 80 countries in 2015 [7,8]. These results point to a heterogeneous and preliminary integration of PC in South America with an important need for advancement.

Progress in PC necessarily involves scientific development. Despite the significant increase in the number of publications on PC in recent decades, the worldwide contribution of South America investigators on the subject remains limited [9,10]. A study presented in 2005 at the European Association of Palliative Care (EAPC) Congress showed that only 0.14% of the publications on PC indexed in Medline had investigators from Latin America [11].

Several strategies can be used to increase scientific production and to improve its quality. Among such strategies is the stimulus for research collaborations with international research centers, especially from locations with greater scientific expertise [12]. The number of articles with international co-authorship has increased significantly since the 1990s in all scientific fields [13]. It is believed that international collaborations increase the potential impact of publications, producing significantly more scientific citations [14,15,16]. Although bibliometric studies on the topic of PC have been published, both with regional and global analyses [9,17,18,19,20], the impact of international research collaborations has not been adequately assessed in the context of PC.

A research collaboration can be defined as two or more researchers working together to produce scientific knowledge; this knowledge is generally measured by the number and quality of the resulting journal publications. In the present study, we are specifically interested in international research collaborations. Our aim was to evaluate the percentage of South America journal articles in the last 20 years involving international research collaborations and the impact of these collaborations on scientific potential and on the number of citations.

## Methods

### Study type

A systematic literature review with bibliometric analysis.

### Ethical aspects

The present analysis is part of a larger study evaluating barriers to conducting research on PC in South America and mapping the scientific production in PC within the region in the last 20 years (Los PAmPAS Study; approved by the Research Ethics Committee of Barretos Cancer Hospital under number 1704/2018).

### Search strategy

Articles published in the period from January 1, 1998, to December 31, 2017, were identified using the keywords (MeSH terms) “palliative care,” “hospice care,” “hospices” and “terminal care,” combined with the geographic location of the following South America countries: “Brazil,” “Argentina,” “Chile,” “Peru,” “Colombia,” “Ecuador,” “Paraguay,” “Uruguay,” “Venezuela” and “Bolivia.” The present work is part of a larger study named Los Pampas which will compare the scientific production of South American countries. Thus, to facilitate furthers comparisons, the authors decided to include in the search strategy only the names of countries with more than 1 million inhabitants. The search was conducted in the electronic databases Pubmed, Embase, Lilacs, and Web of Science (WOS) (Supplementary Material). An experienced librarian participated in the development of the search strategies.

All articles published in peer-reviewed journals, whose main objective was to evaluate PC-related aspects, which had at least one author with an affiliation to a South America country, and which were written in English, Portuguese or Spanish, were included for analysis. If the author was originally from South America but had an affiliation listed in the article with an institution in another country, the article was excluded. Articles that were incomplete or were presented in abstract form at events, those that did not involve humans, and those whose main objective was a disease-modifying treatment, such as surgery, radiotherapy or chemotherapy, were excluded.

In addition to the bibliographic search, a manual search was performed by author name; each first author retrieved in the original search was searched individually in the same databases used for the initial search.

### Data management

The articles retrieved were included in an EndNote library to exclude duplicates. Following the removal of duplicates, the final list of eligible articles to be included in the literature review was analyzed by two reviewers. Disagreements were resolved by a third reviewer.

### Data extraction

A data extraction template was developed to extract all data in REDCap (Research Electronic Data Capture). The following characteristics were extracted from the articles: year of publication, journal name, current PubMed indexation, current impact factor in WOS, current impact factor in Scopus, study design (clinical trial, systematic review or longitudinal study), research external funding (yes vs. no) and PC topic (cancer vs. noncancer). The countries of affiliation and place of work of the investigators were categorized as described in the article. Articles with international research collaboration were considered to be those that had at least two authors with affiliations in different countries, regardless of whether they were internal or external to South America. This classification was categorized into international collaboration (inside and/or outside South America; yes vs. no), international collaboration inside South America (yes vs. no) and international collaboration outside South America (yes vs. no). Citations of the articles in the WOS, Scopus and Google Scholar databases were extracted in December 2017 in two ways: total number over the period (since publication until December 2019) and in the two years following publication (if the article was published in 2015, for example, citations from 2016 and 2017 were extracted).

### Bibliographic map construction

A co-authorship network map was constructed based on bibliographic data using the VOSviewer software version 1.6.13 [21]. A minimum number of two manuscripts per investigator was used for graphic construction. For the analysis, each co-authorship link received the same weight (full counting method).

### Statistical analysis

Data are presented as medians, 25th and 75th percentiles (p25 and p75), and absolute and relative values. The characteristics of the articles were compared between the groups with and without international collaboration using the chi-square test for qualitative variables and the Mann-Whitney test for quantitative variables. The trend of occurrence of international collaborations over time is presented graphically, and significance was calculated using the chi-square test for trends. The total number of citations and the number of citations in the two years after publication were correlated with the number of partner countries inside and outside South America using the Spearman correlation test. A p-value of <0.05 was considered significant. Statistical analyses were performed using SPSS v.20.0.

### Scientific potential assessment

Since the articles were very heterogeneous in methodological design, it was not possible to evaluate their quality using available study quality assessment tools. Thus, the following scientific potential-related variables were extracted from the articles by two independently reviewers: type of study (systematic review, clinical trial or cohort study [yes/no]); Pubmed indexed journal (yes/no); journal with an impact factor ISI-WOS (yes/no); and description of study funding (yes/no).

## Results

A total of 4063 articles were identified in the electronic search, and further 196 were added by complementary manual search. After applying the inclusion criteria, removing duplicates and reading the titles and abstracts, 959 references were selected. Of this total, 318 documents were still excluded, totaling 641 articles for statistical analysis.

The number of articles on PC published by investigators from South America countries increased considerably over the period analyzed. Of the 10 South America countries analyzed, seven had published articles on PC. Brazil (n = 389), Argentina (n = 118), Chile (n = 85) and Colombia (n = 64) had the highest number of published articles. The median (p25–p75) number of total citations and of citations in the two years after publication were 4 (2–9) and 1 (0–2) for WOS; 9 (3–22) and 2 (0–5) for Google Scholar; and 4 (1–9) and 1 (0–3) for Scopus, respectively.

**Figure 1:**
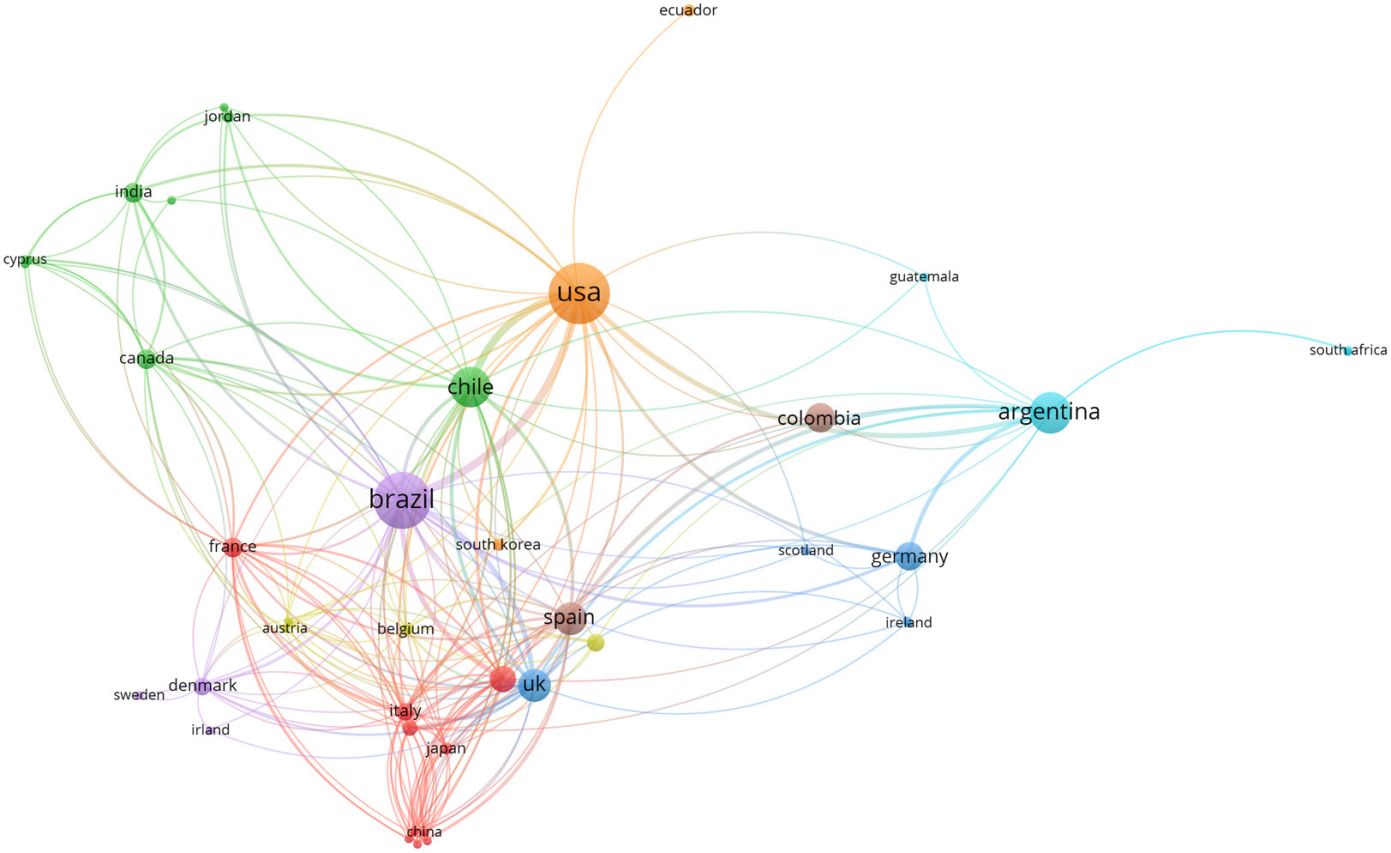
Relationship between the countries involved in international research collaborations. Circle sizes represent number of country publications; line thickness represents number of collaborations between linked countries.

**Figure 2:**
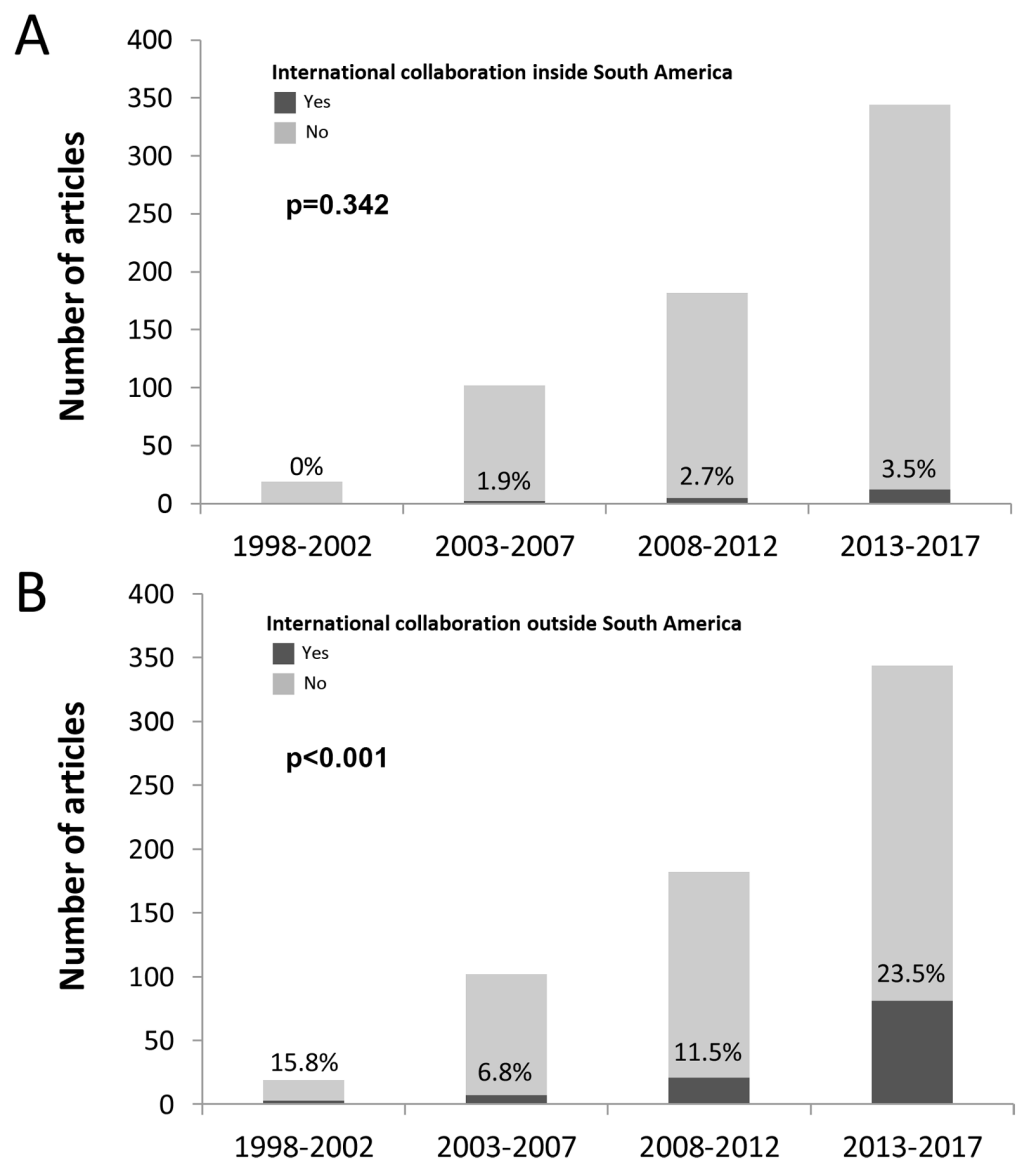
Number and percentage of articles with international collaborations inside South America **(A)** and outside South America **(B)** over the years.

Of the 641 analyzed articles, 117 (18.2%) involved international collaborations; there were 18 (2.8%) and 110 (17.2%) manuscripts involving collaborations between South America countries (internal collaboration) and between countries outside South America (external collaboration), respectively. Among the internal collaborations, 13 (2%), 4 (0.6%), and 1 (0.1%) articles involved collaborations between 2, 3, and 4 countries, respectively. Among the research collaborations outside South America, the number of external countries ranged from 1 (n = 68 articles) to 12 (n = 2 articles). Of the studies with external collaborations (n = 110), 46 (41.8%) had a South America country as the coordinating center (Brazil, n = 21; Argentina, n = 11; Colombia, n = 9; Chile, n = 5). ***Figure 1*** details the relationship between the countries among the international collaborations. ***Figure 2*** shows the trend of occurrence of international collaborations over the years, divided into five-year intervals. Although there was a gradual increase in the percentage of research collaborations inside South America, the chi-square trend analysis was not significant (p = 0.342). However, for international collaborations outside South America, there was an increasing trend over the years (p < 0.001).

Articles involving international collaborations had a higher median number of citations in the two years following publication than did articles without international collaborations (***Table 1***). In addition, international collaborations were associated with greater scientific potential in comparison with publications without international collaborations, as judged by publication in journals indexed in Pubmed (76.1% vs. 41.6%; p < 0.001); impact factor descriptions by WOS (70.1% vs. 29.6%; p < 0.001); studies with described external funding (41.0% vs. 9.7%; p < 0.001); and studies with a clinical trial (5.1% vs. 1.0%; p = 0.002) or cohort design (10.3% vs. 2.9%; p < 0.001). Systematic reviews were not associated with the occurrence of international collaborations (***Table 1***). Supplementary Table 1 shows the associations between citations and characteristics of publication according with internal and external collaborations.

**Table 1:** Association between international collaborations and article characteristics.


CHARACTERISTICS	INTERNATIONAL COLLABORATION		p VALUE

	YES (n = 117)	NO (n = 524)	

	MEDIAN (p25–p75)^2^		

Citations in two years in WOS	2 (1–6)	1 (0–2)	<0.001^1^

Citations in two years in GS	4.5 (2–10)	2 (0–5)	<0.001^1^

Citations in two years in Scopus	3 (1–6)	1 (0–2)	<0.001^1^

Current Journal IF - WOS	2.75 (1.95–3.38)	1.70 (0.98–2.45)	<0.001^1^

Current Journal IF - SJR	2.18 (0.85–3.29)	0.74 (0.51–1.68)	<0.001^1^

	**N (%)**		

Journal with IF - WOS			<0.001^2^

Yes	82 (70.1)	155 (29.6)	

No	35 (29.9)	369 (70.4)	

PubMed indexed journal			<0.001^2^

Yes	89 (76.1)	218 (41.6)	

No	28 (23.9)	306 (58.4)	

Study with funding			<0.001^2^

Yes	48 (41.0)	51 (9.7)	

No	69 (59.0)	473 (90.3)	

Systematic review			0.494^3^

Yes	4 (3.4)	11 (2.1)	

No	113 (96.6)	513 (97.9)	

Randomized Clinical Trial			0.002^2^

Yes	6 (5.1)	5 (1.0)	

No	111 (94.9)	519 (99.0)	

Cohort			<0.001^2^

Yes	12 (10.3)	15 (2.9)	

No	105 (89.7)	509 (97.1)	

Qualitative			<0.001^3^

Yes	6 (5.1)	152 (29.0)	

No	111 (94.9)	372 (71.0)	

Specific to oncology			0.046^2^

Yes	44 (37.6)	148 (28.2)	

No	73 (62.4)	376 (71.8)	


Abbreviations: p25 = 25th percentile; p75 = 75th percentile; WOS = Web of Science; GS = Google Scholar, IF = impact factor; SJR = Scimago Journal Rank. ^1^ Mann-Whitney U Test. ^2^ Chi-square test. ^3^ Fisher exact test.

There were positive correlations of a small magnitude between the number of partner countries inside South America and the number of citations in two years according to Google Scholar, Scopus and WOS (***Table 2***). Correlations of small to moderate magnitude were observed between the number of partner countries outside South America and the number of citations in two years according to Google Scholar (Rho = 0.291, p < 0.001), Scopus (Rho = 0.402, p < 0.001) and WOS (Rho = 0.419, p < 0.001).

**Table 2:** Correlation between numbers of countries involved in international research collaborations and two year citation metrics.


CITATION METRICS	NUMBERS OF COUNTRIES INVOLVED IN RESEARCH COLLABORATION			

	INSIDE SOUTH AMERICA		OUTSIDE SOUTH AMERICA	

	*RHO*	*p-VALUE*	*RHO*	*p-VALUE*

Google Scholar	0.117	0.003	0.291	<0.001

Scopus	0.167	0.001	0.402	<0.001

WOS	0.166	0.001	0.419	<0.001


Legend: WOS = Web of Science.

**Figure 3:**
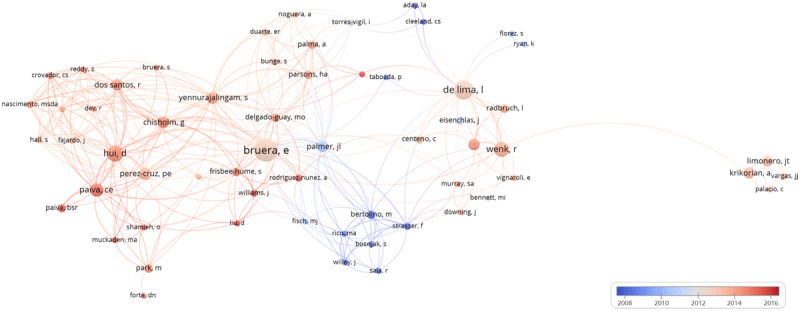
Bibliographic map of co-authorship from publications on Palliative Care including at least on author from South America. Each author is represented by a circle, with the size of the circle representing number of publications. The closer, the greater the relationship between them. The colors represent the years the articles were published.

***Table 3*** shows the ranking of the 15 top articles according to the number of citations (total) in WOS over the analyzed period. Of these, eight are studies with international collaborations, all with MD Anderson Cancer Center (Tx, USA); four of these also had international collaborations inside South America. The bibliographic map (see ***Figure 3***) points to similar results.

**Table 3:** The 15 top articles according to the number of total citations in Web of Science over the analyzed period.


RANKING	TOTAL CITATIONS			TYPE OF IRC	JOURNAL	YEAR	SA COUNTRY	STUDY COORDINATING GROUP

	WOS	SCOPUS	GS					

1	109	155	231	IRC-IO	Journal of Clinical Oncology	2004	Chile, Argentina, Colombia, Brazil	MDACC (Tx, USA)

2	99	128	192	IRC –O	Palliative Medicine	2000	Argentina	MDACC (Tx, USA)

3	98	134	194	No IRC	Journal of Pain and Symptom Management	2006	Argentina	University of Buenos Aires (Argentina)

4	64	70	80	IRC-IO	Cancer	2015	Brazil, Chile	MDACC (Tx, USA)

5	54	55	86	No IRC	BMC Medical Research Methodology	2014	Brazil	Barretos Cancer Hospital (Brazil)

6	54	61	66	IRC-O	JAMA Oncology	2015	Chile	MDACC (Tx, USA)

7	51	60	77	IRC-O	Journal of Clinical Oncology	2013	Chile	MDACC (Tx, USA)

8	50	64	86	IRC-IO	Journal of Pain and Symptom Management	2004	Colombia, Argentina, Chile	MDACC (Tx, USA)

9	46	66	572	No IRC	Pediatric Critical Care Medicine	2003	Argentina	Hospital de Pediatría JP Garrahan, Buenos Aires (Argentina)

10	46	52	101	No IRC	Psycho-Oncology	2012	Chile	PUC-Chile, Santiago (Chile)

11	46	51	73	No IRC	International Journal of Palliative Nursing	2000	Argentina	University of Buenos Aires (Argentina)

12	36	37	65	IRC-O	Journal of Pain and Symptom Management	2015	Brazil	MDACC (Tx, USA)

13	36	46	554	No IRC	Pediatric Critical Care Medicine	2005	Brazil	PUC-RS (Porto Alegre)

14	35	44	69	IRC-IO	The Oncologist	2014	Brazil, Chile	MDACC (Tx, USA)

15	34	46	95	IRC-O	Psycho-Oncology	2012	Colombia	Universidad Pontificia Bolivariana, Medellín (Colombia)


Legend: IRC = international research collaboration; IRC-I = international research collaboration inside South America; IRC-O = international research collaboration outside South America; IRC-IO = international research collaboration both inside and outside South America; MDACC = MD Anderson Cancer Center; Tx = Texas; USA = United States of America; PUC-Chile = Pontificia Universidad Católica de Chile; PUC-RS = Pontíficia Universidade Católica do Rio Grande do Sul; WOS = Web of Science; GS = Google Scholar; SA = South American.

## Discussion

In the present study, we identified that 18.2% of the publications from South America investigators involved international research collaborations. Collaborations between South America countries are infrequent in the context of PC, representing only 2.8% of the publications. In contrast, international collaborations with countries outside South America are more frequent (17.2%) and have shown an increasing trend over the last decades. Studies with international research collaborations are cited more frequently and are greater scientific potential than those without international collaborations, as judged by having more complex methodologies (clinical trials and longitudinal studies), receiving external funding and being published in journals with a higher scientific impact.

Although this was not the objective of this study, the increase in scientific publications on PC from South America investigators over the last 20 years is noteworthy. In 2018, a bibliometric analysis of articles on PC investigated global publications from 2001 to 2016 in the WOS database. A total of 7127 articles by authors from 96 countries was analyzed, with a clear increasing trend in scientific publications on PC worldwide during the evaluated period [18]. This corroborates findings from other studies [17,19], including a 2012 publication that evaluated the scientific production on PC by investigators in Latin America [9]. Although there has been an increase in scientific production in the region, it is not known whether the quality of publications has increased over time. In any case, the present study shows for the first time that international scientific collaborations are important for improving the scientific potential and impact of research on PC.

The trend of economic and social globalization, as well as the ease of connections through the Internet, facilitate the establishment of research partnerships and international collaboration networks. International collaborations can have countless purposes, which may be distinct even for the collaborators within the same study. Multicenter studies have greater power of generalization and greater ability to obtain large sample sizes, sometimes necessary and unfeasible in single-center studies. Furthermore, research collaborations often occur as a function of the knowledge or skills demonstrated by specific researchers as well as the availability of equipment or resources that can implement specific advances in the study. In turn, research collaborations certainly have an educational impact when research centers with less expertise collaborate with large research centers that, consequently, provide capacity building so that the centers in development can attract new researchers and produce higher quality research.

Lack of funding is certainly one of the barriers to conducting research on PC. In the United States of America, for example, less than 1% of government funding for research is directed to topics relevant to PC [22]. Although any biomedical study requires some level of funding, we focused on external funding, which is generally obtained in a very competitive way. Although only 15% of the evaluated studies described having research external funding, the number of studies with funding was more than six fold greater when there was international collaboration outside South America. Research centers under development in South America countries need to stimulate local and regional scientific and technological development in order to increase the amount and quality of scientific production as well as the development of new products with the generation of patents and related products. For this purpose, courses focused on training in international scientific writing and obtaining funding are suggested, in addition to making time available for PC professionals to conduct research [23]. In addition, funding agencies need to direct specific resources to PC and stimulate studies in the field with international collaborations.

A bibliometric review of published articles on supportive and palliative oncology worldwide showed that only 6% of the literature corresponded to randomized clinical trials [20], and even these publications had deficiencies in methodological quality [24]. In our study, less than 10% of all analyzed studies were systematic reviews, clinical trials or prospective studies. In addition, approximately 25% were qualitative studies, and just under two-thirds were published in journals indexed in PubMed. These data indicate that despite the evident increase in scientific production in South America, the quality of this production probably still requires improvement. In terms of citations, a recent publication identified an average of 4 citations (total) per PC article worldwide in WOS [18]. Some countries had articles with a mean number of citations considerably higher than the world average, such as Norway (21.8), Italy (16.9) and Switzerland (16.0). We clearly observed that articles with international collaborations are associated with higher citation rates. Moreover, there was a moderate positive correlation between the number of collaborating countries and the number of citations in two years in WOS.

The present study has several limitations. Since the affiliations were based on what was described in the manuscripts, it is possible that the investigator went to another country as a postdoctoral student, for example, but still has their primary affiliation in South America. The search strategy included keywords related to the geographic region (country names), as for a previous study on Latin America [9]. However, our search was unable to identify all articles and the manual search strategy conducted by author name, initially designed only to validate the search, was necessary to expand the results. Another limitation is how the quality of the studies was evaluated. Ideally, each study should have been evaluated based on its ability to generate changes in the practice of PC. Moreover, the articles could have been evaluated according to methodological quality using tools already accepted worldwide [25]. However, in view of the heterogeneity of the studies, we chose to evaluate their quality indirectly by classifying their methodological design, presence of funding and characteristics of the journal in which the article was published, which is more related to its complexity and scientific potential than necessarily to its quality.

## Conclusions

Scientific production by South America investigators has increased over the last two decades; however, most articles have characteristics of low scientific potential. The number of international collaborations between countries inside South America is smaller than with countries outside South America. Studies with international collaborations, both internal and external to South America, are more frequently cited and have characteristics of greater scientific potential than do studies without international collaborations.

## Additional Files

The additional files for this article can be found as follows:

10.5334/aogh.3158.s1 Supplementary Material. Search strategies.

10.5334/aogh.3158.s2 Supplementary Table 1. Association between international collaborations and article characteristics.
