# Supplementary material for: The Impact of International Research Collaborations on the Citation Metrics and the Scientific Potential of South American Palliative Care Research: Bibliometric Analysis
Source: Ann Glob Health. 2021 Mar 31;87(1):32. doi: 10.5334/aogh.3158 (PMC8015710; doi:10.5334/aogh.3158)
Supplement: Supplementary Material. — Search strategies. [file agh-87-1-3158-s1.pdf]

Supplementary Material. Search strategies

Strategy for Pubmed

(Palliative Care[mh] OR Palliative Care[tw] OR Care, Palliative[tw] OR Therapy, Palliative[tw] OR Palliative Therapy[tw] OR Palliative Treatment[tw] OR Palliative Treatments[tw] OR Treatment, Palliative[tw] OR Treatments, Palliative[tw] OR Palliative Surgery[tw] OR Surgery, Palliative[tw] OR Terminal Care[tw] OR Terminal Care[mh] OR Care, Terminal[tw] OR End of Life Care[tw] OR Care End, Life[tw] OR Care Ends, Life[tw] OR Life Care End[tw] OR Life Care Ends[tw] OR Hospices[tw] OR Hospices[mh] OR Hospice[tw] OR Hospice Care[tw] OR Hospice Care[mh] OR Care, Hospice[tw] OR Hospice Programs[tw] OR Hospice Program[tw] OR Program, Hospice[tw] OR Programs, Hospice[tw] OR Bereavement Care[tw] OR Care, Bereavement[tw]) AND (Brazil[mh] OR Brazil[tw] OR Brasil[tw] OR Uruguay[mh] OR Uruguay[tw] OR Uruguai[tw] OR Argentina[mh] OR Argentina[tw] OR Chile[mh] OR Chile[tw] OR Colombia[mh] OR Colombia[tw] OR Ecuador[mh] OR Ecuador[tw] OR Equador[tw] OR Paraguay[mh] OR Paraguay[tw] OR Paraguai[tw] OR Bolivia[mh] OR Bolivia[tw] OR South America[mh] OR South America[tw])

Strategy for EMBASE

('Palliative Care' OR 'Palliative Care' OR 'Care, Palliative' OR 'Therapy, Palliative' OR 'Palliative Therapy' OR 'Palliative Treatment' OR 'Palliative Treatments' OR 'Treatment, Palliative' OR 'Treatments, Palliative' OR 'Palliative Surgery' OR 'Surgery, Palliative' OR 'Terminal Care' OR 'Terminal Care' OR 'Care, Terminal' OR 'End of Life Care' OR 'Care End, Life' OR 'Care Ends, Life' OR 'Life Care End' OR 'Life Care Ends' OR Hospices OR Hospices OR Hospice OR 'Hospice Care' OR 'Hospice Care' OR 'Care, Hospice' OR 'Hospice Programs' OR 'Hospice Program' OR 'Program, Hospice' OR 'Programs, Hospice' OR 'Bereavement Care' OR 'Care, Bereavement') AND (Brazil OR Brazil OR Brasil OR Urugay OR Urugay OR Urugai OR Argentina OR Argentina OR Chile OR Chile OR Colombia OR Colombia OR Ecuador OR Ecuador OR Equador OR Paraguay OR Paraguay OR Paraguai OR Bolivia OR Bolivia OR 'South America' OR 'South America')

Strategy for LILACS

(mh:(Palliative Care) OR tw:(Palliative Care) OR tw:(Care, Palliative) OR tw:(Therapy, Palliative) OR tw:(Palliative Therapy) OR tw:(Palliative Treatment) OR tw:(Palliative Treatments) OR tw:(Treatment, Palliative) OR tw:(Treatments, Palliative) OR tw:(Palliative Surgery) OR tw:(Surgery, Palliative) OR mh:(Terminal Care) OR tw:(Terminal Care) OR tw:(Care, Terminal)

OR tw:(End of Life Care) OR tw:(Care End, Life) OR tw:(Care Ends, Life) OR tw:(Life Care End) OR tw:(Life Care Ends) OR mh:(Hospices) OR tw:(Hospices) OR tw:(Hospice) OR tw:(Hospice Care) OR mh:(Hospice Care) OR tw:(Care, Hospice) OR tw:(Hospice Programs) OR tw:(Hospice Program) OR tw:(Program, Hospice) OR tw:(Programs, Hospice) OR tw:(Bereavement Care) OR tw:(Care, Bereavement)) AND (mh:(Brazil) OR tw:(Brazil) OR tw:(Brasil) OR mh:(Urugay) OR tw:(Urugay) OR tw:(Urugai) OR mh:(Argentina) OR tw:(Argentina) OR mh:(Chile) OR tw:(Chile) OR mh:(Colombia) OR tw:(Colombia) OR mh:(Ecuador) OR tw:(Ecuador) OR tw:(Equador) OR mh:(Paraguay) OR tw:(Paraguay) OR tw:(Paraguai) OR mh:(Bolivia) OR tw:(Bolivia) OR mh:(South America) OR tw:(South America))

Strategy for Web Of Science

(Palliative Care OR Palliative Care OR Care, Palliative OR Therapy, Palliative OR Palliative Therapy OR Palliative Treatment OR Palliative Treatments OR Treatment, Palliative OR Treatments, Palliative OR Palliative Surgery OR Surgery, Palliative OR Terminal Care OR Terminal Care OR Care, Terminal OR End of Life Care OR Care End, Life OR Care Ends, Life OR Life Care End OR Life Care Ends OR Hospices OR Hospices OR Hospice OR Hospice Care OR Hospice Care OR Care, Hospice OR Hospice Programs OR Hospice Program OR Program, Hospice OR Programs, Hospice OR Bereavement Care OR Care, Bereavement) AND (Brazil OR Brazil OR Brasil OR Urugay OR Urugay OR Urugai OR Argentina OR Argentina OR Chile OR Chile OR Colombia OR Colombia OR Ecuador OR Ecuador OR Equador OR Paraguay OR Paraguay OR Paraguai OR Bolivia OR Bolivia OR South America OR South America)
