# Supplementary material for: The Impact of International Research Collaborations on the Citation Metrics and the Scientific Potential of South American Palliative Care Research: Bibliometric Analysis
Source: Ann Glob Health. 2021 Mar 31;87(1):32. doi: 10.5334/aogh.3158 (PMC8015710; doi:10.5334/aogh.3158)
Supplement: Supplementary Table 1. — Association between international collaborations and article characteristics. [file agh-87-1-3158-s2.pdf]

Supplementary Table 1. Association between international collaborations and article characteristics.

Characteristics	International collaboration inside South America		p value	International collaboration outside South America		p value
	Yes (n=18)	No (n=623)		Yes (n=110)	No (n=531)	
	Median (p25–p75)[2]			Median (p25–p75)[2]		
Citations in two years in WOS	3 (1.75–10)	1 (0–2)	0.001[1]	2.5 (1–6)	1 (0–2)	<0.001[1]
Citations in two years in Google Scholar	5 (2.5–11.5)	2 (0–5)	0.003	5 (2.50–10)	2 (0–5)	<0.001[1]
Citations in two years in Scopus	3 (1–13)	1 (0–3)	0.001[1]	3 (1–6)	1 (0–2)	<0.001[1]
Current Journal IF - WOS	4.96 (3.38–6.10)	1.92 (1.01–2.75)	<0.001[1]	2.75 (1.96–3.38)	1.69 (0.98–2.45)	<0.001[1]
Current Journal IF - SJR	3.29 (0.56–5.46)	0.89 (0.53–1.90)	0.008[1]	2.18 (1.03–3.29)	0.74 (0.51–1.66)	<0.001[1]
	N (%)			N (%)		
Journal with IF - WOS			0.031[2]			<0.001[2]
Yes	11 (61.1)	397 (63.7)		81 (73.6)	156 (29.4)	
No	7 (38.9)	226 (36.3)		29 (26.4)	375 (70.6)	
PubMed indexed journal			0.015[3]			<0.001[2]
Yes	14 (77.8)	293 (47.0)		85 (77.3)	222 (41.8)	
No	4 (22.2)	330 (53.0)		25 (22.7)	309 (58.2)	
Study with funding			0.142[2]			<0.001[2]
Yes	5 (27.8)	94 (15.1)		47 (42.7)	52 (9.8)	
No	13 (72.2)	529 (84.9)		63 (57.3)	479 (90.2)	
Systematic review			1.000[3]			0.323[2]
Yes	0 (0.0)	15 (2.4)		4 (3.6)	11 (2.1)	
No	18 (100.0)	608 (97.6)		106 (96.4)	520 (97.9)	
Randomized Clinical Trial			0.002[2]			0.001[2]
Yes	2 (11.1)	9 (1.4)		6 (5.5)	5 (0.9)	

No	16 (88.9)	614 (98.6)		104 (94.5)	526 (99.1)	
Cohort			<0.001[2]			0.001[2]
Yes	5 (27.8)	22 (3.5)		11 (10.0)	16 (3.0)	
No	13 (72.2)	601 (96.5)		99 (90.0)	515 (97.0)	
Qualitative			0.010[3]			<0.001[3]
Yes	0 (0)	158 (25.4)		6 (5.5)	152 (28.6)	
No	18 (100)	465 (74.6)		104 (94.5)	379 (71.4)	
Specific to oncology			0.016[2]			0.038[2]
Yes	10 (55.6)	182 (29.2)		42 (38.2)	150 (28.1)	
No	8 (44.4)	441 (70.8)		68 (61.8)	381 (71.8)	

Abbreviations: p25 = 25th percentile; p75 = 75th percentile; WOS = Web of Science; GS = Google Scholar.
[1]Mann-Whitney U Test. [2]Chi-square test. [3]Fisher Exact Test.
